# Increased tumor necrosis factor-α, cleaved caspase 3 levels and insulin receptor substrate-1 phosphorylation in the β_1_-adrenergic receptor knockout mouse

**Published:** 2011-07-06

**Authors:** Surekha Rani Panjala, Youde Jiang, Timothy S. Kern, Steven A. Thomas, Jena J. Steinle

**Affiliations:** 1 Department of Ophthalmology, Hamilton Eye Institute, The University of Tennessee Health Science Center, Memphis, TN; 2 Department of Anatomy and Neurobiology, The University of Tennessee Health Science Center, Memphis, TN; 3 Departments of Medicine and Ophthalmology, Case Western Reserve University, Cleveland, OH; 4 Department of Pharmacology, University of Pennsylvania, Philadelphia, PA

## Abstract

**Purpose:**

To investigate the role of β1-adrenergic receptors on insulin like growth factor (IGF)-1 receptor signaling and apoptosis in the retina using β1-adrenergic receptor knockout (KO) mice.

**Methods:**

Western blotting and enzyme-linked immunosorbent assay analyses were done on whole retinal lysates from β1-adrenergic receptor KO mice and wild-type littermates. In addition, vascular analyses of degenerate capillaries and pericyte ghosts were done on the retina of the β1-adrenergic receptor KO mice versus littermates.

**Results:**

Lack of β1-adrenergic receptors produced a significant increase in both degenerate capillaries and pericyte ghosts. This was accompanied by an increase in cleaved caspase 3 and tumor necrosis factor α levels. IGF-1 receptor phosphorylation was not changed; however, protein kinase B (Akt) phosphorylation was significantly decreased. The decrease in Akt phosphorylation is likely caused by increased insulin receptor substrate-1 serine 307 (IRS-1^Ser307^) phosphorylation, which is inhibitory to IGF-1 receptor signaling.

**Conclusions:**

These studies further support the idea that maintenance of β-adrenergic receptor signaling is beneficial for retinal homeostasis. Loss of β1-adrenergic receptor signaling alters tumor necrosis factor α and apoptosis levels in the retina, as well as Akt and IGF-1 receptor phosphorylation. Since many of these same changes are observed in the diabetic retina, these data support that novel β-adrenergic receptor agents may provide additional avenues for therapeutics.

## Introduction

Diabetic retinopathy is the leading cause of blindness in working-age adults; however, other than optimal glucose control, few therapies exist for the early stages of the disease. Over the past several years, numerous hypotheses have been put forward as to the causes of the retinal damage common to diabetic retinopathy, including oxidative stress, advanced glycation end-products, inflammatory factors, altered protein kinase C signaling, etc. [1-4]. In addition to these hypotheses, we have reported that loss of sympathetic neurotransmission produces changes in the retina similar to diabetic retinopathy [5-7]. We have reported that loss of dopamine β-hydroxylase (*Dbh^−/−^*) produces retinal damage similar to retinopathy [6]. We found that insulin-like growth factor (IGF)-1 receptor signaling was reduced in the *Dbh^−/−^* mice, leading to increased apoptosis [8]. These data suggested that loss of norepinephrine signaling could alter IGF-1 receptor signaling and apoptosis in the retina. It is likely that these changes occur in retinal endothelial cells, since endothelial cells from other targets are 100× more responsive to IGF-1 than to insulin receptor–mediated signaling [9]. Additionally, we have recently shown that retinal endothelial cells respond to a β1-adrenergic receptor agonist to regulate IGF-1 receptor signaling with minimal changes in the insulin receptor [10]. Additionally, we have found that human retinal endothelial cells only possess β1 and β3-adrenergic receptors, without expressing the β2-adrenergic receptor [11]. We have focused on the β-adrenergic receptors, since we have shown that β-adrenergic receptor antagonists produce deleterious changes in the retina [7], while restoration of β-adrenergic signaling to diabetic rats can prevent the neuronal and vascular changes associated with diabetic retinopathy [12]. While the mechanism of action for isoproterenol has not been established, it was noted that diabetes increased tumor necrosis factor (TNF)α levels, which were reduced with the β-adrenergic receptor agonists. Additionally, isoproterenol reduced cleaved caspase 3 levels [10]. Since we have shown that loss of norepinephrine binding to β-adrenergic receptors in the retina led to decreased IGF-1 receptor levels, we investigated whether IGF-1 receptor signaling cascades are altered in mice without β1-adrenergic receptors. Therefore, we hypothesized that IGF-1 receptor signaling would be reduced in the retina of β1-adrenergic receptor knockout mice, leading to increased apoptosis. While we did observe an increase in apoptosis, it was mediated through TNFα pathways, rather than IGF-1 receptor signaling, in the β1-adrenergic receptor knockout mice.

## Methods

### Animal preparation

Four- to six-month-old wild-type (WT) and β1-adrenergic receptor knockout (KO) mice on a hybrid 129/Sv x C57BL/6 background [13] were generated by mating heterozygotes; genotype was determined by PCR. Retinas from each animal were obtained from Dr. Steven Thomas (University of Pennsylvania). The retina was extracted for protein analyses or whole globes removed for vascular analyses. The eyes from these mice appeared grossly normal and protein amounts from the KO retina were similar to that collected from WT mice. Only one time point was used for all analyses. All animal procedures were approved by the Institute Animal Care and Use Committees of the University of Tennessee Health Science Center and the University of Pennsylvania and followed NIH guidelines.

### Western blot analysis

Western blot analysis was done as described previously [10,12]. Primary antibodies to total IGF-1 receptor (1:500; Cell Signaling, Danvers, MA), phosphorylated IGF-1 receptor (Tyr 1135/1136, 1:500; Cell Signaling), phosphorylated Akt (Ser473, 1:500; Cell Signaling), total Akt (1:500; Cell Signaling), phosphorylated insulin receptor substrate (IRS)-1(Ser307, 1:500; Cell Signaling), total IRS-1 (1:500; Cell Signaling), and IGFBP-3 (1:500; Gro-Pep, Novozymes, Australia) were applied overnight at 4 °C. Secondary antibodies conjugated to horseradish peroxidase were used at 1:5000 (Promega, Madison, WI). Western blot membranes were processed using a Kodak image station 4000 MM system (Carestream Health, Rochester, NY). Using Prism software (GraphPad software, San Diego, CA), mean densitometry numbers or the ratio of mean densitometry of phosphorylated protein to total protein were used to compare data from β1-adrenergic knockout (KO) and control (WT) mice using Mann–Whitney tests, with p<0.05 being accepted as significant. Data are expressed as mean densitometry in arbitrary units (A.U.) and in the case of phosphorylated protein, data are expressed as a ratio of phosphorylated protein levels to total protein levels in arbitrary units.

### Enzyme-linked immunosorbent assay analyses

Enzyme-linked immunosorbent assay (ELISA) analyses for cleaved caspase 3 (Cell Signaling) and TNFα (Pierce, Rockford, IL) were performed according to the manufacturer’s instructions. The ELISA analyses were done in pre-coated 96-well plate format. Standards were added to the first row, while samples loaded in triplicate were added to the remaining wells. Following overnight incubation, plates were washed and secondary antibody was added. After the specified time, plates were washed and the colorimetric reagent was added to the plate. Plates were read on a spectrophotometer. An equal amount of protein was loaded for each sample, so results are reported as the optical density of the mean± standard error of the mean (SEM) for the β1-adrenergic receptor KO and WT mice.

### Measurement of degenerate capillaries and pericyte ghosts

For the acellular capillary and pericyte ghost counts, retinas from five mice each of the β1-adrenergic receptor^−/−^ and control animals were used. Eyes were enucleated and placed into 10% buffered formalin for 5 days. The retina was dissected in 3% crude trypsin solution (Difco Bacto Trypsin 250; Difco, Detroit, MI) containing 0.2 M sodium fluoride at 37 °C for 2 h [14]. The neural retina was gently brushed away and the remaining retinal vascular tree was dried onto a glass slide.

Once the isolated retinal vascular tree was dried onto the glass slide, the slide was stained with hematoxylin-periodic acid-Schiff. Degenerate (acellular) capillaries were counted in the midretina in six to seven fields evenly spaced around the retina. Degenerate capillaries were identified as capillary-sized tubes with no nuclei anywhere along their length. Degenerate capillaries were counted only if their average diameter was at least 20% of that found in surrounding healthy capillaries. Pericyte ghosts were estimated from the prevalence of spaces in the capillary basement membranes from which pericytes had disappeared. The number of pericyte ghosts was determined in multiple midretinal fields, and is reported per 1,000 capillary cells [15,16]. All measurements were done in a masked manner.

## Results

### Increased degenerate capillaries and pericyte ghosts in β1-adrenergic receptor knockout mice

**Figure 1:**
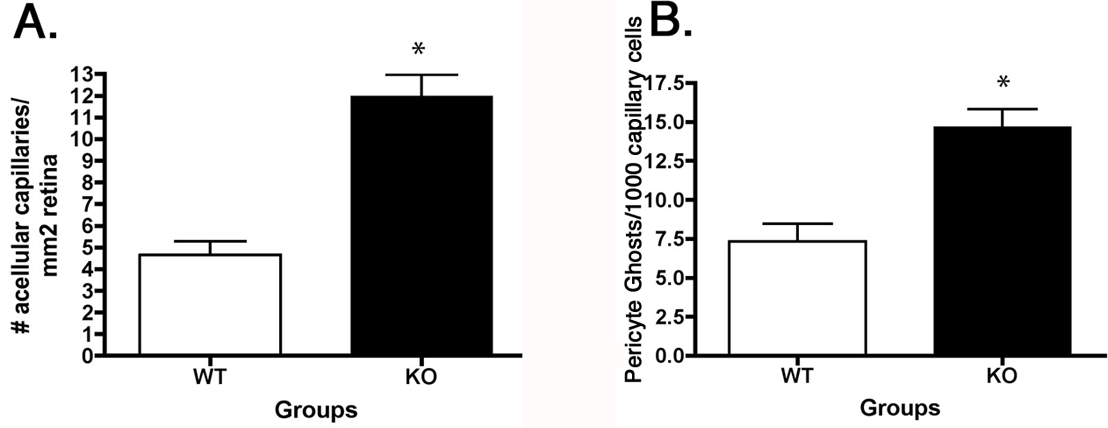
Degenerate capillaries and pericyte ghosts in the β1-adrenergic receptor retina **A**: This panel is a bar graph of the number of degenerate capillaries in the β1-adrenergic receptor knockout (KO) mice versus wild-type (WT) littermates. **B**: This panel is a bar graph of the number of pericyte ghosts in the same samples of retina from β1-adrenergic receptor KO and their WT littermates. *p<0.05 versus WT. n=5 for each group.

We have previously published that dopamine-β-hydroxylase KO mice have increased numbers of degenerate capillaries and pericyte ghosts [6], while treatment with topical isoproterenol, a β-adrenergic receptor agonist, can reduce degenerate capillary formation in the rat retina [12]. We focused on the vascular changes in this study as retinal endothelial cells possess β1-adrenergic receptors and likely undergo apoptosis. We wanted to determine whether the loss of β1-adrenergic receptor signaling was involved in the cell death noted by the formation of degenerate capillaries and pericyte ghosts. Indeed, we found a threefold increase in degenerate capillary formation and a twofold increase in pericyte ghost formation in the β1-adrenergic receptor KO mice when compared to their WT littermates (p<0.05 versus WT, Figure 1).

### Cleaved caspase 3 levels are increased in whole retinal lysates from β1-adrenergic receptor knockout mice

**Figure 2:**
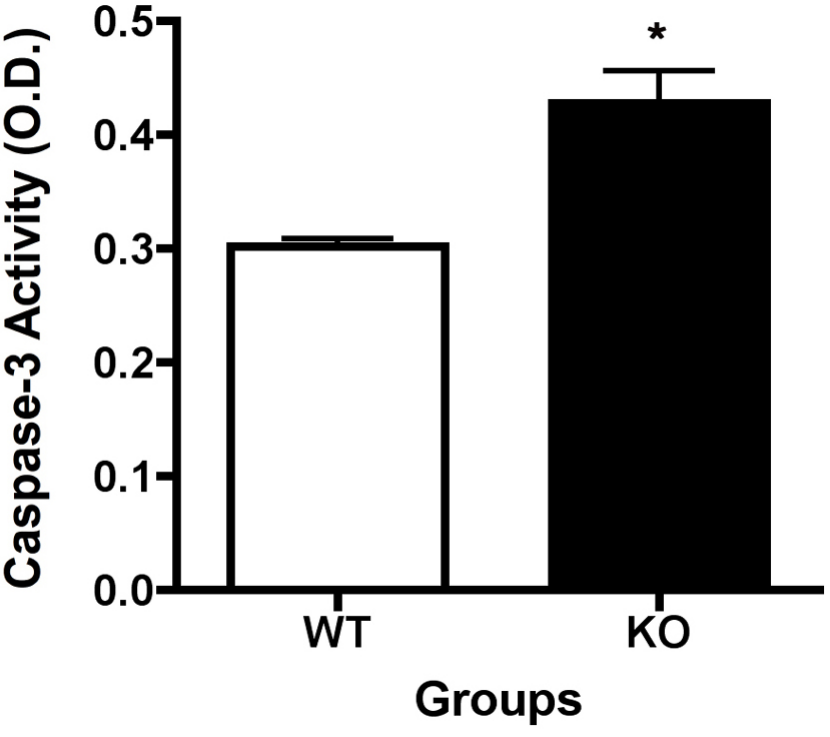
Cleaved caspase 3 is increased in knockout animals. Enzyme-linked immunosorbent assay (ELISA) analyses of whole retinal lysates from β1-adrenergic receptor knockout (KO) animals versus their wild-type (WT) littermates. Caspase 3 levels are increased in the knockout mice KO animals. *Significance was found at p<0.05 versus wildtype samples. Animal numbers (N) is equal to 6.

Since we saw the increased numbers of pericyte ghosts and degenerate capillaries in the β1-adrenergic receptor KO mice, we measured levels of cleaved caspase 3, as a marker of apoptosis. We found a significant increase in the cleavage of caspase 3 in the β1-adrenergic receptor KO animals, as compared to WT mice (p<0.05 versus WT, Figure 2).

### No changes in insulin like growth factor-1 receptor (IGF-1R) receptor levels between the β1-adrenergic receptor knockout and wild-type mice

**Figure 3:**
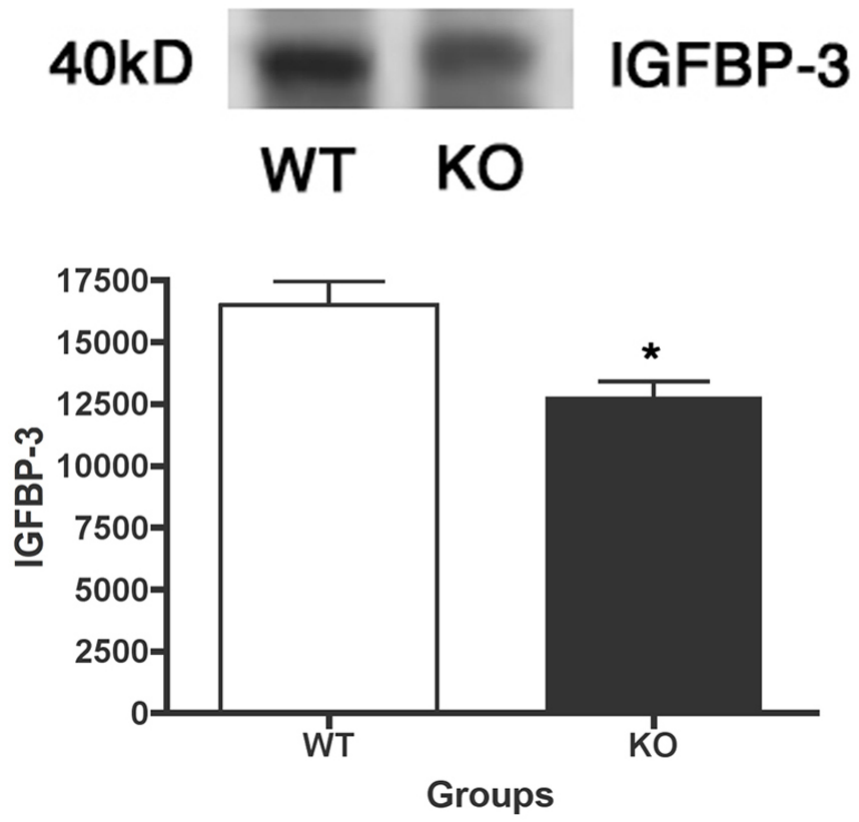
Retina from β1-adrenergic receptor has reduced insulin-like growth factor binding protein (IGFBP)-3 levels. Representative blots and bar graph of IGFBP-3 protein levels in whole retinal lysates in the β1-adrenergic receptor knockout (KO) mice and their wild-type (WT) controls. *Significance was found at p<0.05 versus wildtype samples. Animal numbers (N) is equal to 6.

**Figure 4:**
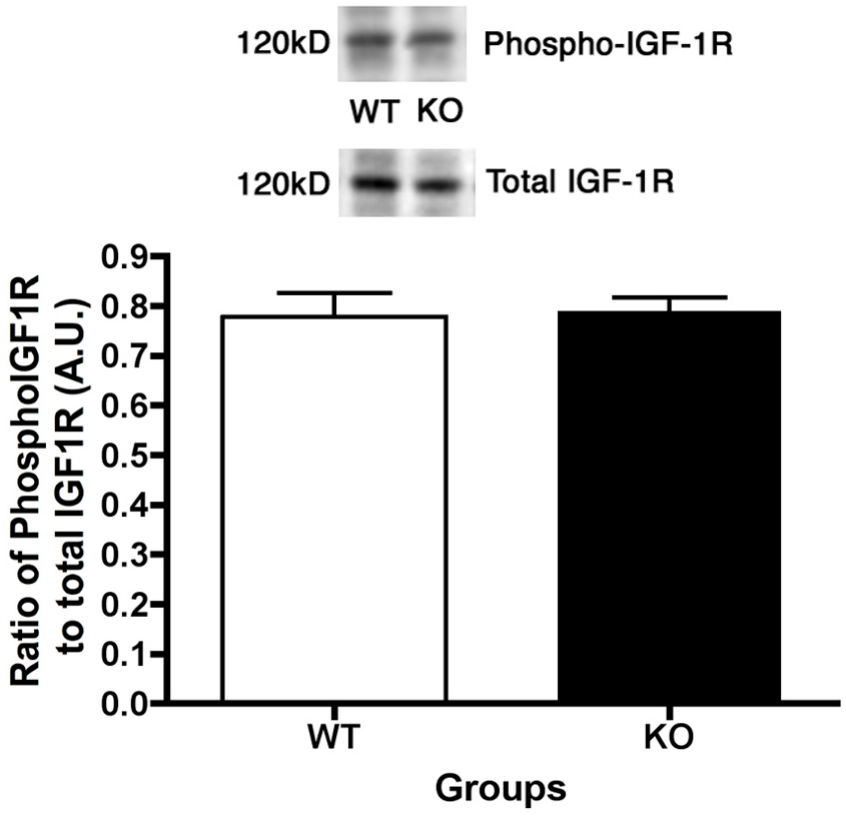
Insulin like growth factor-1 (IGF-1) receptor phosphorylation does not change in β1-adrenergic receptor knockout retina. Representative blots and bar graph of phosphorylated IGF-1 receptor (Tyr 1135/1136) and total IGF-1 receptor protein levels in retinal lysates in the β1-adrenergic receptor knockout (KO) mice and the wild-type (WT) controls. Bar graph is the ratio of phosphorylated protein to total protein levels. *Significance was found at p<0.05 versus wildtype samples. Animal numbers (N) is equal to 6.

We recently published that IGF-1R phosphorylation is significantly decreased in *Dbh^−/−^* mice. We therefore expected that IGF-1R phosphorylation would also be decreased in mice lacking β1-adrenergic receptors. Additionally, we have recently shown that the β-adrenergic receptor antagonist propranolol produced a significant decrease in insulin-like growth factor binding protein (IGFBP)-3 levels [7], which would decrease the amount of IGF-1 bound to IGFBP-3 in the circulation, thus allowing more IGF-1 to bind its receptor. Despite the reduced IGFBP-3 levels in the retina of β1-adrenergic receptor KO mice (p<0.05 versus WT, Figure 3), we found that IGF-1R phosphorylation is not altered in retinal lysates from β1-adrenergic receptor knockout mice compared to their littermates (Figure 4).

### Despite no changes in IGF-1R phosphorylation, Akt phosphorylation is reduced

**Figure 5:**
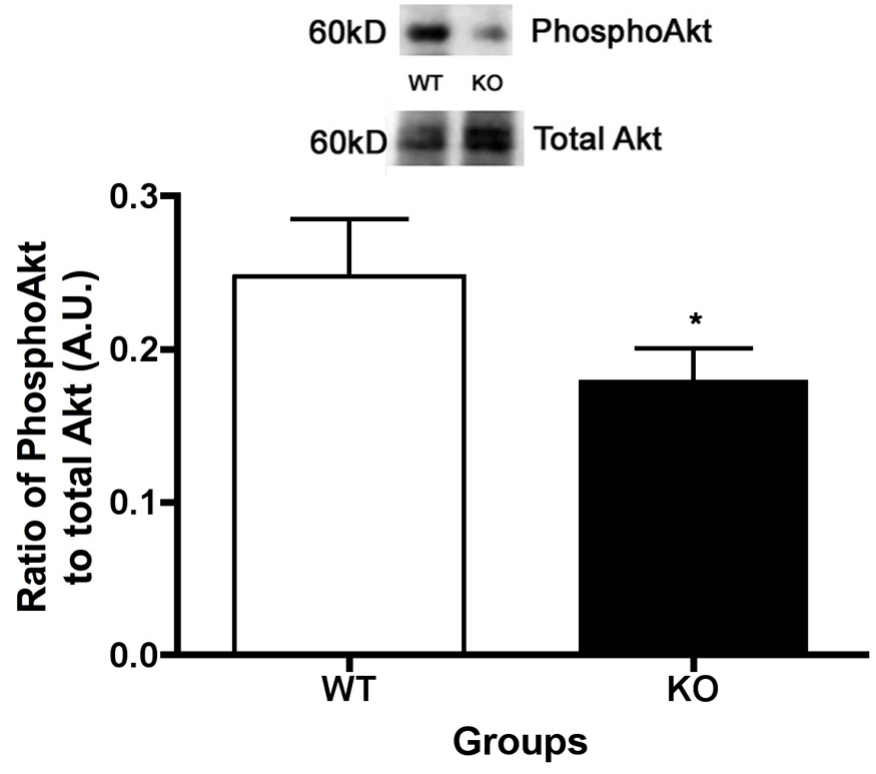
Despite no change in insulin like growth factor-1 receptor phosphorylation, Akt phosphorylation is reduced in retina from β1-adrenergic receptor knockout mice. Representative blots and bar graph of the ratio of phosphorylated Akt (Ser 473) to total Akt protein levels in retinal lysates in the β1-adrenergic receptor knockout (KO) mice and the wild-type (WT) controls. *p<0.05 versus WT. n=5.

The typical cellular signaling cascade for the IGF-1R leads to increased phosphorylation of Akt, producing reduced apoptosis, which we have shown previously in retinal endothelial cells [10], in the retina of rats treated systemically with propranolol [7], and in dopamine β-hydroxylase KO mice [8]. Since we did not see changes in IGF-1R phosphorylation, the cascade would predict that Akt should not be changed, despite the loss of β1-adrenergic receptors. However, the phosphorylation of Akt is reduced in the β1-adrenergic receptor KO mice (p<0.05 versus WT, Figure 5). These data for Akt could explain the increase in cleaved caspase 3 levels, since Akt typically inhibits the cleavage of caspase 3. However, it is unclear why Akt phosphorylation is reduced in mice with no changes in IGF-1R phosphorylation.

### TNFα levels are increased in the β1-adrenergic receptor knockout mice

**Figure 6:**
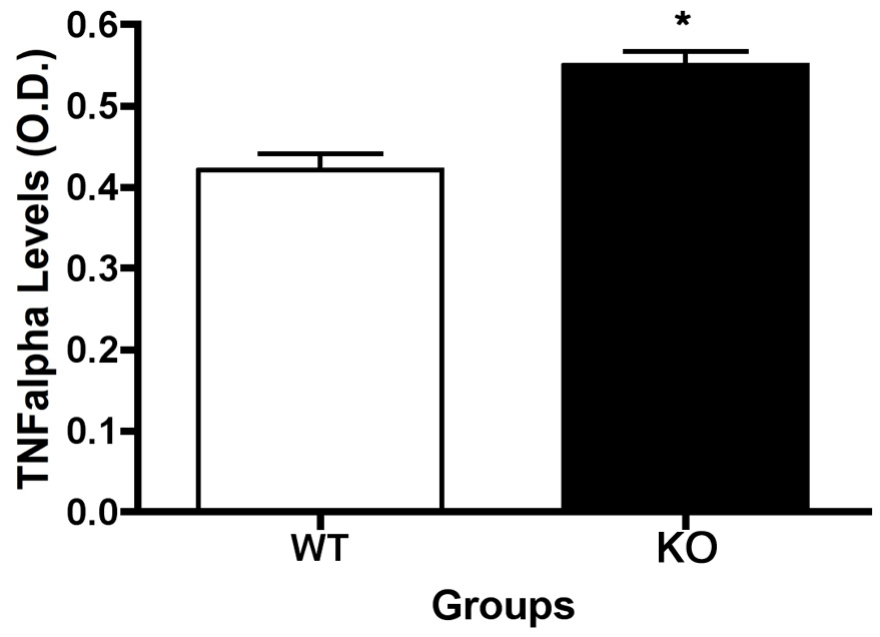
Enzyme linked immunosorbent assay results demonstrate that lack of β1-adrenergic receptors increases tumor necrosis factor a levels. Significance demonstrates that p<0.05 versus wildtype samples. Seven animals in each group were used for these studies.

In addition to the increased cleavage of caspase 3, a significant increase in TNFα levels were observed in the β1-adrenergic receptor KO mice (p<0.05 versus WT, Figure 6). This follows our previous findings of reduced TNFα levels in diabetic animals treated with β-adrenergic receptor agonists [12], and in retinal Müller cells cultured in high glucose [17].

### Increased IRS-1^Ser307^ in β1-adrenergic receptor knockout mice, potentially due to increased TNFα levels

**Figure 7:**
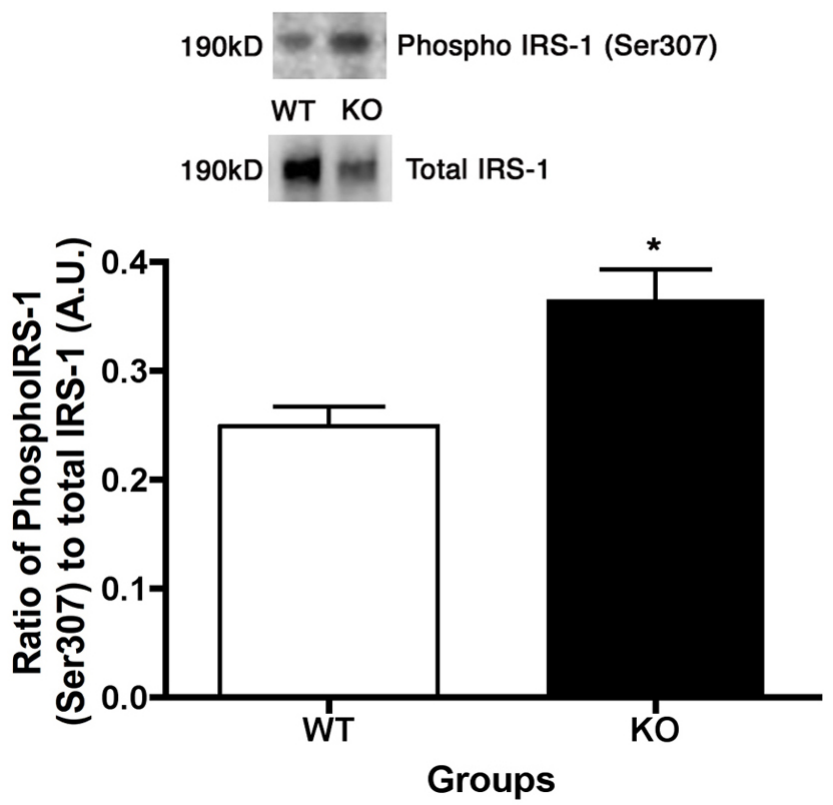
Insulin receptor substrate-1 serine 307 (IRS-1^Ser307^) is increased in retinal lysates from β1-adrenergic receptor knockout mice. Western blot image and bar graph of the ratio of phosphorylated IRS-1^Ser307^ to total IRS-1 in the retina of β1-adrenergic receptor knockout (KO) mice versus wild-type (WT) littermates. *p<0.05 versus WT, n=4.

Work on adipocytes has suggested that increased TNFα levels can produce increased phosphorylation of IRS-1 on serine 307, which is inhibitory to insulin/IGF-1 receptor signaling [18,19]. We have found a similar phenomenon in retinal Müller cells cultured in high glucose (Walker et al., in submission). An increase in IRS-1^Ser307^ would limit the signaling of IGF-1R to Akt, resulting in increased cleaved caspase 3 levels. Indeed, we observed that IRS-1^Ser307^ phosphorylation is significantly increased (46%) in the retina of the β1-adrenergic receptor KO mice as compared to their WT littermates (p<0.05, Figure 7). Taken together, the increased TNFα levels in the β1-adrenergic receptor knockout mice likely decreases Akt activity, producing increased apoptosis in the retina of the β1-adrenergic receptor KO mice.

## Discussion

We hypothesized that IGF-1 signaling would be decreased in the β1-adrenergic receptor KO mouse retina, as we observed in the *Dbh^−/−^* mouse retina. The loss of IGF-1R signaling would increase apoptosis, which could explain the increased numbers of degenerate capillaries and pericyte ghosts. However, we did not observe changes in IGF-1R phosphorylation in the retinal lysates from the β1-adrenergic receptor KO mice. Yet, we did find a significant increase in the cleavage of caspase 3, as well as reduced Akt phosphorylation. Taken together, this suggested that something was different in the cellular signaling of the retina from β1-adrenergic receptor KO mice, compared to retinal samples from *Dbh^−/−^* mice.

Within the laboratory, we have recently found that retinal endothelial cells and retinal Müller cells respond very different to altered β-adrenergic receptor signaling; however, both cell types respond to β-adrenergic receptor stimulation with a reduction in TNFα levels and cleaved caspase 3 [17,20,21]. Based on these findings, we would expect that retinal lysates from β1-adrenergic receptor KO mice would have increased TNFα levels and cleaved caspase 3 levels, which was observed in this study. The question at hand was why are caspase 3 levels increased when IGF-1R phosphorylation is not altered? Furthermore, does TNFα have a direct link to apoptosis in the retina of β1-adrenergic receptor KO mice?

Over the past 15 years or more, the regulation of IGF-1 signaling has become of increasing importance, as many reported that IGF-1 signaling may regulate longevity [22]. IGFBPs comprise a large family of highly regulated proteins originally found to regulate the bioavailability of IGF-I to bind its tyrosine kinase receptor [22]. IGFBPs regulate the delivery of IGF molecules to local tissues [23], with the predominating IGFBP species determined by cell type and local conditions. Important to this study is that IGFBP-3 may play a novel role to prevent endothelial cell apoptosis in an IGF-independent manner [24,25]. The protective effects of IGFBP-3 in primary retinal endothelial cells appear to contrast with reported actions of IGFBP-3 in immortalized cancer cell lines, where it has been well established that IGFBP-3 promotes apoptosis via several IGF-independent mechanisms [26-28]. We have previously reported that propranolol, a β-adrenergic receptor antagonist, leads to a decrease in IGFBP-3 protein levels [7]. In the present study, we find significantly reduced IGFBP-3 protein levels in the β1-adrenergic receptor KO mouse retina, which follows the findings from the work with propranolol. Since others have reported that IGFBP-3 can be antiapoptotic to the retina in the oxygen-induced retinopathy model [25,29], these findings suggest that the increased cleaved caspase 3 observed in the β1-adrenergic receptor KO mice may be due to reduced levels of IGFBP-3 in the retinal endothelial cells, resulting in increased apoptosis through IGF-1 receptor-independent effects.

In addition to the antiapoptotic actions of IGFBP-3, the increase in TNFα levels may also be responsible for the increased cleaved caspase 3 levels in the β1-adrenergic receptor knockout mice. TNFα may regulate apoptosis in one of two ways: through inhibition of insulin/IGF-1 signaling [30,31] or directly through the death receptor pathway. Ultimately, both pathways lead to increased cleaved caspase 3 levels. Based on the increased IRS-1^Ser307^ levels in the β1-adrenergic receptor knockout retinal lysates, it appears more likely that the increased TNFα in the KO animals inhibits any antiapoptotic actions of IGF-1R. This finding has been reported in other cell types, specifically in adipocytes and muscle [18,19]. The increase in IRS-1^Ser307^ could explain why Akt phosphorylation was reduced, despite no change in IGF-1R phosphorylation. Therefore, data suggest that increased TNFα levels inhibit IGF-1R signaling to Akt, which results in increased cleaved caspase 3 levels, as well as increased degenerate capillaries and pericyte ghosts.

While the data suggest that the increased cleaved caspase 3 levels can be induced in two separate ways based on data presented in this study (through decreased IGFBP-3 levels and increased IRS-1^Ser307^ phosphorylation), alterations in insulin signaling could also be involved. In the present study, we did not investigate insulin receptor since we have recently shown that β1-adrenergic receptors have limited actions on insulin receptor phosphorylation [10]. Based on the work by Panjala, insulin receptor phosphorylation would be increased in the β1-adrenergic receptor KO mice, which would inhibit apoptosis. This does not follow the findings in the current study. Additionally, work in other endothelial cells has suggested that endothelial cells have a 100× preference for IGF-1 receptor signaling over insulin receptor signaling [9]. An additional limitation of the present study is that we did not look at changes in the retinal morphology, other than degenerate capillaries. While the eyes from the KO and WT animals were grossly normal and no differences in overall retinal vasculature were noted, measurements of the retina were not obtained. However, when analyses of protein content are considered, no significant differences in total retinal proteins were noted between the β1-adrenergic receptor KO mice and the WT mice. Finally, analyses of multiple time points would be optimal. Unfortunately, in the present study, we only collected retinal samples from one time point in these animals. Future studies may investigate temporal changes in both neural and vascular changes in this mouse model.

Overall, this is the first study of the retinal signaling and vascular morphology in the β1-adrenergic receptor KO animals. Data support work in the surgical sympathectomy and *Dbh^−/−^* mice to demonstrate that loss of sympathetic neurotransmission can produce retinal damage. In addition to finding that β1-adrenergic receptor signaling is involved in maintenance of retinal homeostasis, these findings suggest that increased TNFα levels and/or reduced IGFBP-3 protein levels are involved in the proapoptotic response observed in the retinas from β1-adrenergic receptor KO mice. Future studies will focus on the cellular mechanisms by which β-adrenergic receptors can directly regulate IGFBP-3 and TNFα to prevent retinal damage. Nonetheless, these findings further support the hypothesis that loss of sympathetic neurotransmission is involved in the retinal vascular changes similar to those occurring in diabetic retinopathy, and suggest that maintenance of β-adrenergic receptor signaling may offer a novel therapeutic for retinal disease.
